# Ultra-High-Molecular-Weight-Polyethylene (UHMWPE) as a Promising Polymer Material for Biomedical Applications: A Concise Review

**DOI:** 10.3390/polym12020323

**Published:** 2020-02-04

**Authors:** Muzamil Hussain, Rizwan Ali Naqvi, Naseem Abbas, Shahzad Masood Khan, Saad Nawaz, Arif Hussain, Nida Zahra, Muhammad Waqas Khalid

**Affiliations:** 1 Mechanical Engineering Department, NFC Institute of Engineering and Technology, Multan 60000, Pakistan; muzamilhussain833@gmail.com; 2 Department of Polymer Engineering and Technology, University of the Punjab, Lahore 42000, Pakistan; shahzadmqkhan@hotmail.com; 3 Department of Unmanned Vehicle Engineering, Sejong University, Seoul 05006, Korea; rizwanali@sejong.ac.kr; 4 School of Mechanical Engineering, College of Engineering, Chung-Ang University, 84 Heukseok-ro, Dongjak-gu, Seoul 06974, Korea; 5 Department of Mechanical Engineering, University of Engineering & Technology Lahore, KSK-Campus, Sheikhupura 39350, Pakistan; dr.saadnawaz@uet.edu.pk; 6 Department of Mechanical Convergence Engineering, Hanyang University, Seoul 04763, Korea; ahaengr@gmail.com; 7 Department of Physics, Government College University Faisalabad, Faisalabad 38000, Pakistan; nzahra.phy@gmail.com; 8 Biomedical Engineering Technology Department, NFC Institute of Engineering and Technology, Multan 60000, Pakistan; waqaskhalid15@gmail.com

**Keywords:** ultra-high molecular weight polyethylene (UHMWPE), biomedical materials, tribological performance, coefficient of friction (COF), irradiation, surface modifications

## Abstract

Ultra-High Molecular Weight Polyethylene (UHMWPE) is used in biomedical applications due to its high wear-resistance, ductility, and biocompatibility. A great deal of research in recent decades has focused on further improving its mechanical and tribological performances in order to provide durable implants in patients. Several methods, including irradiation, surface modifications, and reinforcements have been employed to improve the tribological and mechanical performance of UHMWPE. The effect of these modifications on tribological and mechanical performance was discussed in this review.

## 1. Introduction

Ultra-High Molecular Weight Polyethylene (UHMWPE) is an engineering polymer that varies from high-density polyethylene (HDPE) in terms of average molecular weight and average chain length [1]. According to the International Standards Organization (ISO), UHMWPE has a molecular weight of at least 1 million g/mole and degree of polymerization of 36,000, while according to the American Society for Testing and Materials (ASTM) it has a molecular weight of greater than 3.1 million g/mole and degree of polymerization of 110,000 [2]. The properties of UHMWPE are highly dependent on their microstructure rather than molecular mass [3]. UHMWPE is a semi-crystalline polymer that contains fully crystalline and fully amorphous phases as an interfacial all-trans phase [4,5]. In the crystalline phase, the particular lamellar shape of crystallite is due to the chain folding with the chain axis, which enlarges the chain fold area. In the amorphous phase, the chains are interconnected through occasional crosslinks and random entanglements instead of proper chain folding. The relation between amorphous and crystalline phases are provided by tie molecules. The crystallinity of UHMWPE depends on its volumetric percentage of crystallites [6]. The properties of UHMWPE are determined by the connections between amorphous and crystalline phases, i.e., tie molecules, crystallinity, the degree of crosslinks and entanglements; and the positions of the crystallites. The average properties of UHMWPE and HDPE are presented in Table 1. 

UHMWPE has high wear-resistance, toughness, durability, and biocompatibility. Therefore, it is commonly used as a bearing material with ceramic or metallic counter surfaces in joint arthroplasty [8,9] UHMWPE’s significance for achieving outstanding performance in total joint arthroplasties is unquestionable [10,11]. For long-term clinical applications, its tribological performance and lifetime are key aspects [12,13]. However, UHMWPE implants have limited life due to their wear complications. When the UHMWPE is used in the periprosthetic environment it induces osteolysis followed by loosening of the implant. This implant loosening is joined with fatigue causes the aseptic loosening which ultimately causes the implant’s failure. [14,15,16,17]. Many methods such as improving cross-linking [18,19,20,21], or crystallinity percentage [22,23,24,25] through irradiation [26], surface modification through plasma treatment [27,28], or introducing effective textures [29,30], and reinforcements with particles or fibers [31,32,33] have been used for enhancing properties of UHMWPE.

Few review articles [2,18,34] have been published to correlate the mechanics and morphology of UHMWPE with its wear and mechanical properties. In one review [35], the influence of CNT and graphene as reinforcements for UHMWPE is evaluated. In a few review articles [3,36], other advances in UHMWPE for improving wear and mechanical performance are discussed. However, in such articles, many studies on other polymeric materials are considered for supporting the evidence and there is a lack of clarity regarding the optimal values of the effective methods. The objective of this study is to summarize the existing practices for the enhanced tribological and mechanical performance of UHMWPE. The influence of irradiation, reinforcements and surface modifications is briefly discussed and a tabular data is presented for estimating the optimal values or materials. As a conclusion, by using the UHMWPE, mechanical and tribological findings were further improved in order to provide durable implants in patients. 

## 2. Irradiation 

Crosslinking of UHMWPE significantly improves wear performance [37,38,39,40], which can be achieved through the use of a silane [41,42], or chemical methods using peroxides [38,43] and irradiation [44,45,46]. The free radicals produced by these methods create the inter-chain covalent bonds, leading to the formation of crosslinking. Moreover, these long-lived free radicals react with oxygen, resulting in a cascade of different reactions [47]. The overall free oxidation mechanism is a chain reaction that involves polymer chain scission and produces different end products such as carboxylic acids and ketones [48]. This oxidation reduces the mechanical performance of UHMWPE [49,50]. The reduction in properties is associated with molecular weight and cross-link density. 

### 2.1. Crosslinking and Crystallinity

**Figure 1:**
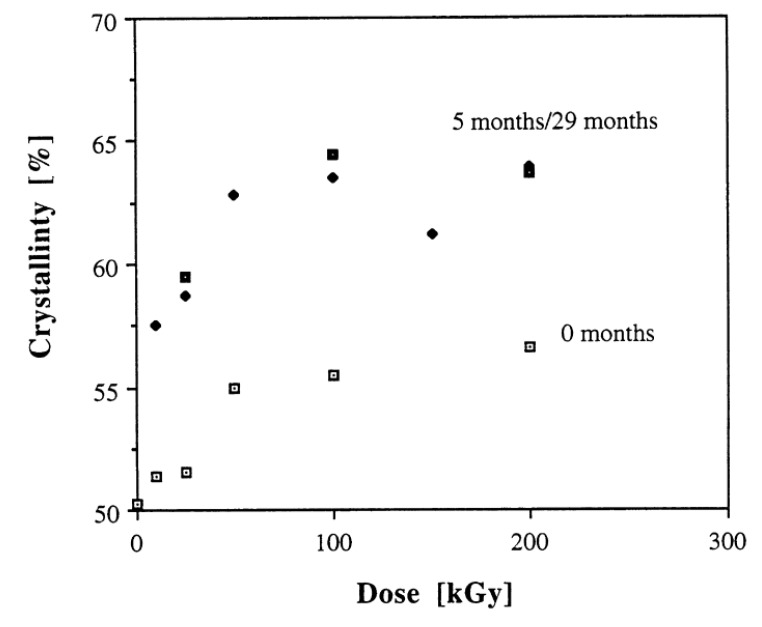
Crystallinity variation with dose and time. Reprinted with permission from [60].

**Figure 2:**
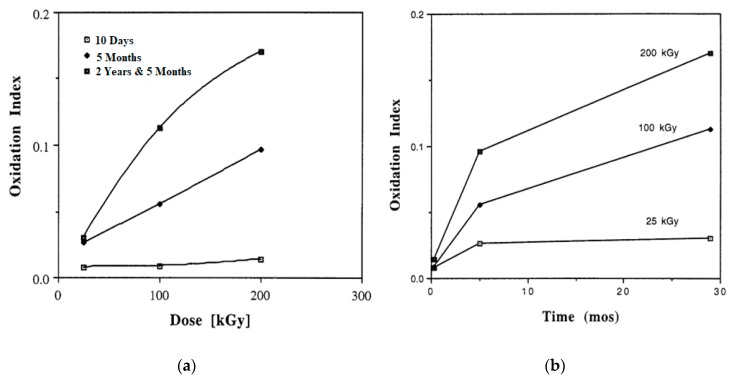
The change in oxidation index with (**a**) radiation dose; and (**b**) aging time. Reprinted with permission from [60].

Among all methods of crosslinking, irradiation is the most common and effective method for sterilizing and/or crosslinking UHMWPE [45,51,52,53]. However, the irradiation produces free radicals in UHMWPE and the trapped radicals decay slowly in it [52]. The decay of free radicals is important, as it provides information about the overall reaction mechanism in the presence of oxygen. Along with crosslinking, the formation of transvinylene units and chain scission are the common processes in UHMWPE in the result of irradiation. The transvinylene content and crosslink density increased at a higher radiation dose [54]. The irradiated component with higher transvinylene contents showed a higher oxidation rate. The level of oxidation can be assessed through the content of transvinylene units [54,55]. After irradiation, the chain-folded crystallization and recrystallization occur in UHMWPE in the presence of crosslinks. These changes in chain folding kinetics, result in decreased crystallinity [56,57,58]. Since the reduced crystallinity allows oxygen to diffuse deeper into the UHMWPE through the amorphous region. Additionally, the allylic hydrogens at trans-vinylene bonds are easier to extract than the hydrogens at tertiary alkyl carbons. These factors combined with induced strain energies facilitate the oxidation mechanism, probably by reducing the energy barrier for chain scissions reaction at more reactive sites. Fung showed [59] a relation between initial transvinylene content and maximum oxidation. The critical oxidation levels were determined for gamma and e- beam treatments at different radiation doses. It was found that the oxidation levels were highly dependent on radiation dose for both sources. The increase in ketone oxidation index with irradiation dose in terms of loss in mechanical properties is observed. Premnath [60] irradiated UHMWPE specimens in the air with electrons and then these were aged at room temperature for different times to investigate the alterations in molecular rearrangements and micro-molecular structure. The crystallinity of UHMWPE for several radiation doses, and for different time intervals are shown in Figure 1. The increase in crystallinity with increasing irradiation dose was observed, probably due to the rearrangement of chains at the amorphous domain following the chain scission of molecules in this interface. The plot of absorbed dose in terms of oxidation index and irradiation time is presented in Figure 2a,b. The oxidation index with dose was almost in a linear manner at all times whereas increment in oxidation index was higher at starting time interval as compared to higher times. The oxidation varies approximately linearly with dose because of the linear variations in free radicals concentration with dose. The decrease in oxidation rate was probably due to the diffusion of free radicals from the crystalline region to the amorphous domain and/or diffusion of oxygen from the amorphous interface to the radical along with crystal stalks; and/or reaction kinetics of different oxidation reactions. Karuppiah [22] investigated the effect of crystallinity on the wear performance of UHMWPE and found that the scratch depth and friction force tended to decrease with increasing the crystallinity. Their study suggested that the wear resistance can be increased with increasing the degree of crystallinity. 

Multiple factors influenced the crystallinity and oxidative degradation by irradiation [61,62]. The dose and dose rate of irradiation strongly influence the crystallinity and oxidation of UHMWPE [55,63,64,65,66,67,68,69]. A suitable post-irradiation process eliminates free radicals to prevent degradation of UHMWPE over the long period and to promote the stability against oxidation. [18,55,59]. Subjecting UHMWPE to a subsequent below-melt annealing or remelting step reduces radicals and the degree of oxidation. The UHMWPE chains can be fold and the crystalline lamellae can be formed by heating the UHMWPE at high pressure and cooling it above the melting temperature. The resulting crystallinity of UHMWPE is increased after the formation of a crystalline structure [70,71,72].

Other different parameters, such as temperature [73,74], packaging atmosphere and packaging [75,76,77], processing conditions [78], also influence the distribution and the amount of the oxidation products. In general, the irradiation at high temperatures, low dose rates and in the presence of oxygen enhance the oxidation process, which strongly degrades the UHMWPE. Bracco [45] analyzed gamma sterilized prosthetic components to study the effect of implant packaging materials and temperature. Three groups of packaging materials including multilayer polymeric barrier packaging, gas permeable packaging, and a combination of polymeric and metallic foils packaging were used. The concentration of oxygen and alkyl macro-radicals was assessed by FTIR analysis. The ROOH are more important than carbonyl in oxidation because ROOH are the first oxidation products. The hydroperoxides/ketones concentration was low for first two packaging groups, while was very high for third packaging group. The difference in concentration is due to the different oxygen permeability of packaging materials. The rate of decomposition was proportional to local temperature during sterilization. It is concluded that the negligence in selection of irradiation parameters can cause the unpredictable oxidation and degradation of UHMWPE. 

### 2.2. Aging

**Figure 3:**
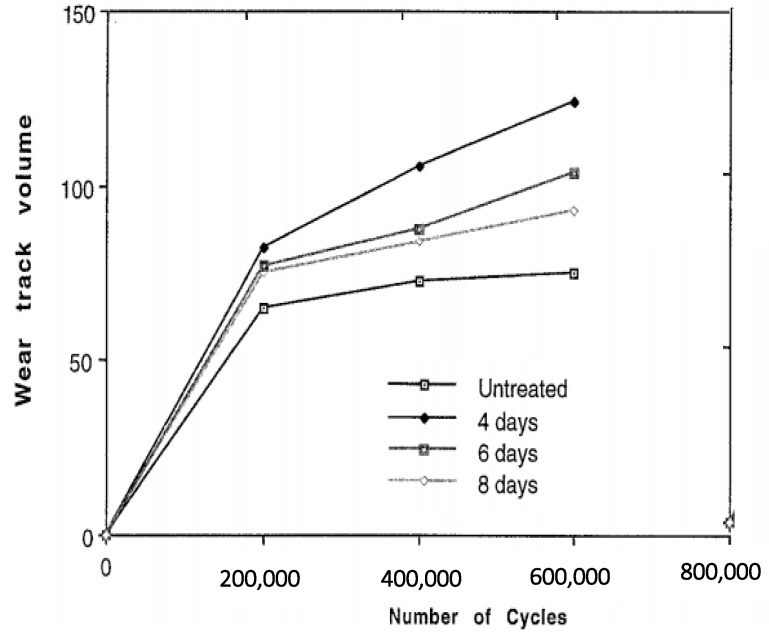
Change in wear volume under sliding tests for unaged and aged specimens. Reprinted with permission from [86].

With aging, the reaction of trapped free radicals with oxygen enhances due to the deep diffusion of oxygen and causes more oxidation. The active free radicals in UHMWPE undergo the intramolecular and intermolecular decompositions resulting in the time-dependent chain scission. This process gradually reduces the crosslinking in the aged UHMWPE. With this, the tie chain scission process allows growth to occur and, further crystal perfection thus the aged UHMWPE has higher crystallinity. The oxidation index increases due to the thickening of the oxidized surface layer with an increase in aging time. In addition, the irradiation of aged samples showed low crosslinking as well as higher oxidation [79,80,81,82,83,84]. This shows crosslinking and oxidation both are highly dependent on aging. The variations in the level of cross-linking, crystallinity, and oxidation during aging cause the change in mechanical properties. The brittleness in the aged UHMWPE liners enables the production of cracks under sliding shear and tensile stress states and eventually, it can enhance the wear of UHMWPE. Lee [85] compared the wear performance of un-irradiated and gamma-irradiated UHMWPE specimens and studied the effect of aging. The wear was measured in terms of weight loss. The wear of gamma-irradiated specimens was lower than un-irradiated specimens and it was increased with aging time. However, the oxidation index of un-irradiated specimens was lower than irradiated specimens. To investigate the influence of oxidation on wear the specimens were artificially aged for 2 to 8 days and tested at knee simulator under sliding conditions. Bell [86] investigated the influence artificially induced subsurface on the wear of UHMWPE. Figure 3 shows the change in wear track volume for untreated and aged (oxidized) specimens. The large volumetric change at the initial stage for all specimens is attributed to the creep of the UHMWPE [87].

**Figure 4:**
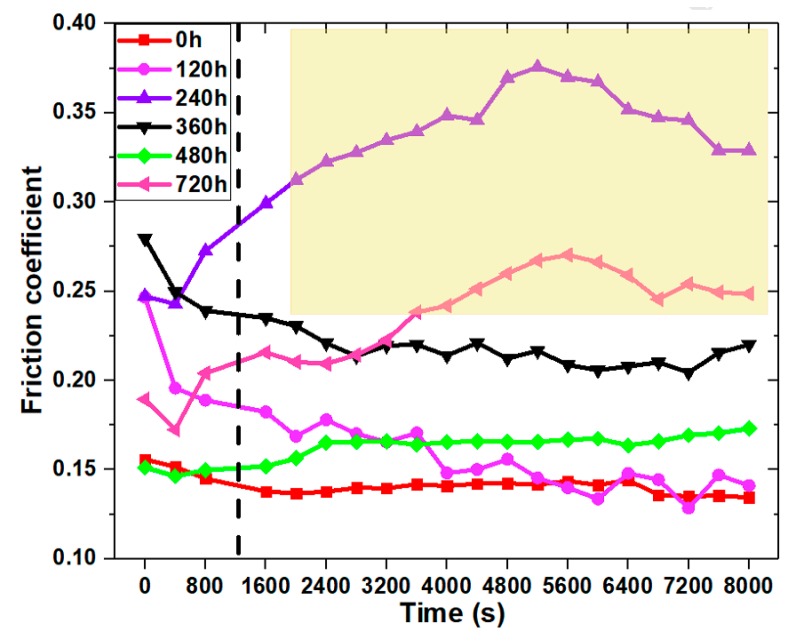
The coefficient of friction (COF) of UHMWPE for different aging times. Reprinted with permission from [88].

Chang [88] investigated the tribological performance of aged UHMWPE specimens and confirmed the obvious influence of aging conditions on wear and mechanical performance. The coefficient of friction of aged UHMWPE specimens for different aging periods is shown in Figure 4. The coefficient of friction (COF) was increased up to 65.96% for 720 h aging time and 80 °C aging temperature. It was found that the tribological and mechanical degradation was attributed to the damage in the molecular structure of UHMWPE. 

### 2.3. Wear and Mechanical Degradation Mechanism 

**Figure 5:**
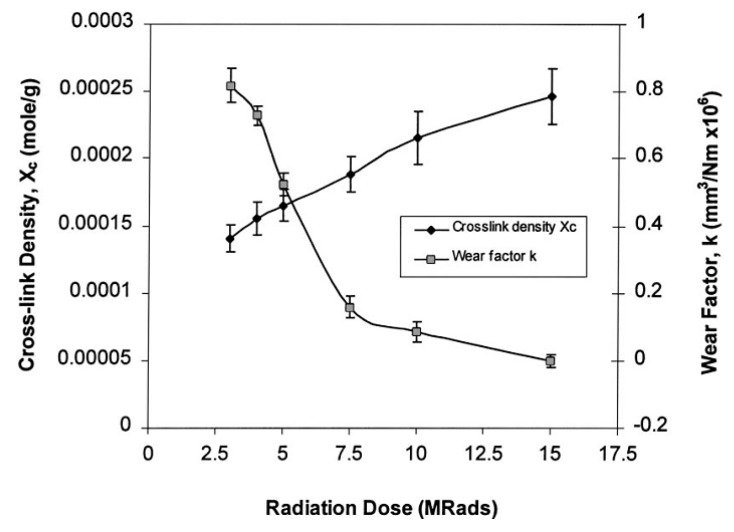
The COF of UHMWPE for different aging times. Reprinted with permission from [94].

Delamination is the catastrophic type of wear in UHMWPE bearing components [3,86]. The diffusion of free radicals out to polymer matrix or into the polymer as a result of irradiation can lead to the development of a subsurface oxidized band. This subsurface oxidation region can lead to delamination and in many cases, failure occurs from subsurface crack initiation and propagation [4]. Bell [86] investigation on retrieved total knee implants has shown that oxidation of UHMWPE can be influenced by stresses induced during everyday activities or by post-irradiation associated with the subsurface band. This study showed that the delamination occurred only in the presence of the subsurface oxidized band and propagated through this band. In wear test, an increase in oxidation produced increased surface wear without delamination. Similarly, in fatigue and tensile tests, there was a reduction in the fatigue resistance and in ultimate tensile strength of oxidized UHMWPE specimens. Oxidation increased the fatigue crack growth rate. It was also observed that the resistance to oxidation was different in different grades of UHMWPE. The wear mechanism of UHMWPE can be better understood by treating it anisotropic material [2,89]. The strength of UHMWPE depends on the direction in which load is applied. So a wear mechanism can be better assessed by multidimensional wear tests [90]. Wang [91] performed hip-joint simulator experiments on both linear and crosslinked UHMWPE to investigate the effect of molecular chain orientation on the wear surfaces and within wear debris. The UHMWPE specimens sterilized by ethylene oxide gas in the air and by gamma irradiation in nitrogen were used for tests. Crosslinking was not achieved by ethylene oxide gas sterilization. Results obtained from the hip simulator test indicated that the wear resistance of UHMWPE can be significantly improved by radiation-induced cross-linking. The strength of bearing surfaces in multidirectional sliding experiments was lower than the bearing surfaces in uniaxial tensile tests. The phenomenon of strain-softening in UHMWPE bearing surfaces is also due to the structural anisotropy. This study recommends maintaining the homogenous and isotropic molecular structure of UHMWPE bearing surfaces for achieving high résistance to strain to harden. A large number of wear models have been developed to explain the morphology of wear debris [84,92,93]. Wang [94] proposed a theoretical model for UHMWPE based on the concept of frictional work under multi-directional lubricated sliding conditions. Based on the theory, the wear volume loss per unit load and sliding distance was related to the cross-link density, COF, and the maximum shear angle. The COF and wear volume rate of UHMWPE were decreased with increasing the cup/head clearance. The linear increase in wear volume loss was observed with an increased COF. The wear rate was shown to increase linearly with increasing the COF. The wear rate can be decreased by irradiation as indicated by results in hip simulator. The effect of radiation dose on crosslinking and wear factor is shown in Figure 5. The linear increase in wear rate was observed with increasing the molecular weight into crosslinks. 

The mechanical degradation of UHMWPE is very important for high stressed bearing components which may cause large deformations or fatigue damage such as or delamination or pitting. The multiaxial deformation of UHMWPE is more important than the deformation under uniaxial loading conditions for clinical relevance. After irradiation UHMWPE deforms a spatially non-uniform towards a more brittle (less ductile) behavior. Edidin [95] investigated the mechanisms of mechanical degradation of UHMWPE, including both the linear and non-linear responses, as a function of aging. An increase in elastic modulus and a decrease in work to failure, ultimate displacement and ultimate load as a result of accelerated and natural aging were demonstrated in this study. 

The influence of irradiation on the level of crystallinity, tribological performance and mechanical performance reported in the literature is presented in Table 2. The parameters and their values in percentage are presented as compared to pure UHMWPE. 

All results reported in Table 2 show that the optimal value for radiation dose is in the range of 25–50 kGy in terms of less oxidation and high tribological properties. The variation in optimal value suggests that the selection of radiation dose depends upon the several conditions discussed in previous sections. So careful selection of the amount of radiation dose is mandatory. The significant difference in gel content percentage and crosslink density percentage can be observed for the mentioned results. The increase in the crystallinity percentage is in the range of 5 to 26%. The values of the oxidation index and transvinylene index are increased from 10–125% and 2–12% respectively as compared to pure UHMWPE. Several mechanical properties such as toughness, elongation at break, impact strength, ultimate displacement, ultimate load, and ultimate displacement are decreased, while hardness and tensile strength are increased or maintained. 

### 2.4. Methods for Minimizing Degradation

It is well established that irradiation results in the mechanical degradation of UHMWPE [59,66,76,98,99]. Therefore, it needs to focus on methods to enhance crosslinking by maintaining mechanical properties. Muratoglu [100] irradiated UHMWPE in the air at high temperature by a high dose-rate electron beam with adiabatic heating and then melted. The wear resistance was improved by this method with maintaining the mechanical performance of UHMWPE for hip implants because of the absence of free radicals and as a result of high oxidation resistance. The three years follow-up showed an equal decrease in wear rate for argon sterilized and air sterilized UHMWPE due to the initial creep after implantation and thereafter wear rate decreased steadily slow. The argon sterilized UHMWPE liners showed more stable due to the less wear rate as compared to air-sterilized liners after nine years follow-up. Despite their different patterns and amounts of wear, no difference in osteolytic tissue reaction is demonstrated [101].

Sterilizing UHMWPE in the oxygen-depleted atmospheres, like vacuum packaging or inert gas, can reduce the degree of oxidative degradation [47,62]. Faris [102] observed less wear in inert-sterilized molded liners than air-sterilized extruded liners after a 6 years follow-up in 150 patients. He concluded that the molded UHMWPE is more resistant to wear than the extruded UHMWPE [103]. Goosen [101] observed a difference in wear rate between the AIR and ARGON liners based on multivariate analysis during a follow-up of 3–12 years. There was no significant difference in wear rate for three years after implantation. Thereafter, the ARGON liner showed a decreased wear rate tan AIR liner.

Bracco [45] postulated that unsaturated additives can be added into UHMWPE to enhance the cross-linking to increase the reactions involving terminal double bonds. UHMWPE specimens soaked in ethylene, methyl-acetylene, and 1,7-octadiene respectively, were irradiated using different doses of an electron beam. Gel fraction results showed that all irradiated samples are crosslinked, and 1,7-octadiene exhibits the most efficient additive for enhancing crosslinking. The mechanical results revealed a significant decrease in ultimate stress and elongation at break with high doses of an electron beam in multiple passages. 

**Figure 6:**
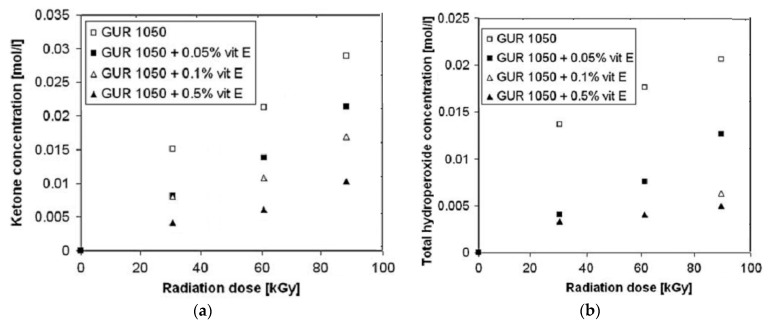
The effect of radiation dose on the amount of oxidation products after 2390 h of aging time (**a**) ketones; (**b**) total hydroperoxides. Reprinted with permission from [99].

Vitamin E has been considered as an important antioxidant to reduce the oxidation and wear degradation of UHMWPE components [104,105]. Vitamin E reacts with trapped free radicals into the UHMWPE, impending them to react with oxygen. Thus, it prevents oxidative degradation of UHMWPE and increases its resistance to wear and fatigue [72,106,107]. Costa [99] investigated the efficiency of vitamin E for stabilizing UHMWPE. UHMWPE powder was blended with pharmaceutical grade vitamin E and consolidated into large slabs. The formation of a stable-tocopheryl radical due to the interaction between macro-alkyl radicals and vitamin E results in a decrease of macro-alkyl radicals. The reactions between macro-alkyl radicals with oxygen can be inhibited due to the decrease in alkyl radicals (which react with vitamin E) and to the vitamin E reaction with peroxy macroradical. The data is shown in Figure 6. Moreover, the crosslinking effectiveness is reduced due to the possibility of the reaction between macro-alkyl radicals with vinyl double bonds, or vitamin E. 

## 3. Reinforcements

An important method to enhance the properties of UHMWPE is the reinforcement with polymers [108]. Many reinforcing materials such as zinc oxide particles, glass, carbon nanoparticles, and others have been employed to improve the wear resistance of UHMWPE. [109,110,111,112,113,114,115,116,117,118,119]. Inorganic particles such as alumina [120], silica [121] or hydroxyapatite [122], have been reported for improving wear and mechanical performance. 

UHMWPE fibers are also considered as high-performance fibers for various applications due to their high mechanical performance with low density [123].

### 3.1. Carbon Particles

**Figure 7:**
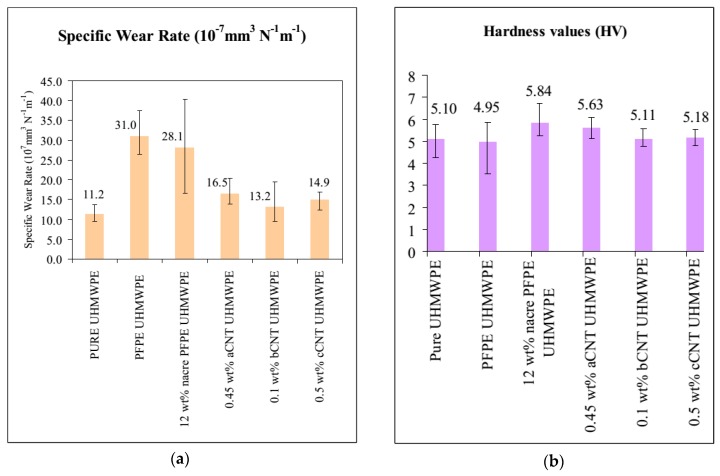
(**a**) Wear rate for different UHMWPE composites (**b**) The Vickers hardness values for different UHMWPE composites. Reprinted with permission from [126].

The carbon nanoparticles such as carbon nanotube (CNT), carbon nanofiber (CNF), graphene, nanodiamonds are used as reinforcing materials to enhance the mechanical properties of UHMWPE and to achieve the long lifetime of implants. Unique optical, electrical, mechanical and thermal properties of CNT and their utilization for making composites have been gained remarkable attention [124]. CNT is an important additive for polymer composites to achieve improved wear resistance [118]. The dispersion of CNT into the polymer materials and interaction between the CNT and macromolecular chains are key factors to transfer the CNT properties to polymeric matrix [125]. Liu [126] added three types of CNTs and nacre into UHMWPE, coated with Perfluoropolyether (PFPE) to study the wear of UHMWPE under lubricated condition. The UHMWPE coated with PFPE and reinforced with nacre showed higher wear than pure UHMWPE. The dispersion of all types of CNTs into UHMWPE also increases the wear rate of UHMWPE. The reason for increasing wear rate is that the non-covalent stresses and merely shear interactions between UHMWPE molecules and CNT and dispersion of CNT into UHMWPE cannot enhance the required energy dissipation to decrease build up the plastic strain. The hardness value decreased by reinforcing PFPE with UHMWPE probably due to softening the surface of UHMWPE and reducing the resistance between the material and indenter surface. The dispersion of nacre into UHMWPE and PFPE coating increase hardness value slightly due to the addition of hard nacre particles. The data is presented in Figure 7.

Golchin [127] investigated the tribological performance of nanodiamond reinforcing particles into UHMWPE under water-lubricated sliding conditions. The dispersion of nanodiamond into UHMWPE significantly reduced friction and wear properties. Due to nanoscale dimensions and semi-spherical morphology of nanodiamond, it acts as bearing balls between the tribo surfaces and the detached wear debris can roll. This improves direct asperity-asperity contacts and reduces the friction between the mating surfaces. The reduction in shear stress acting on the chains of UHMWPE due to the reduced friction force leads to the decrement in wear rate of the tribo-pairs.

Similar to CNT and nanodiamond, CNF has gained considerable attention as a reinforcement for polymers due to their promising intrinsic properties and their good compatibility [128,129]. Galetz [130] dispersed the CNF into the extruded UHMWPE and investigated the mechanical properties. The yield stress and modulus were improved with maintaining the ductility. In addition, the hardness of UHMWPE was increased due to smoothness in the surface provided by the reinforcement of nanoscale particles. Sui [131] added CNF into UHMWPE. The tensile modulus and tensile strength of the UHMWPE/HDPE were increased with the incorporation of CNF, while a decrease appeared at a higher content. Zulkifli [132] studied the effect of inter-ply stacking positions on the mechanical performance of hard ballistic UHMWPE/carbon fibers composites. The significant variations in the back-face signature, flexural yield strength, and ballistic impact were observed by varying the small change in orientation of carbon fibers. The results show that the ballistic performance can be boosted by a strategic sequence of the carbon fibers in UHMWPE. 

**Figure 8:**
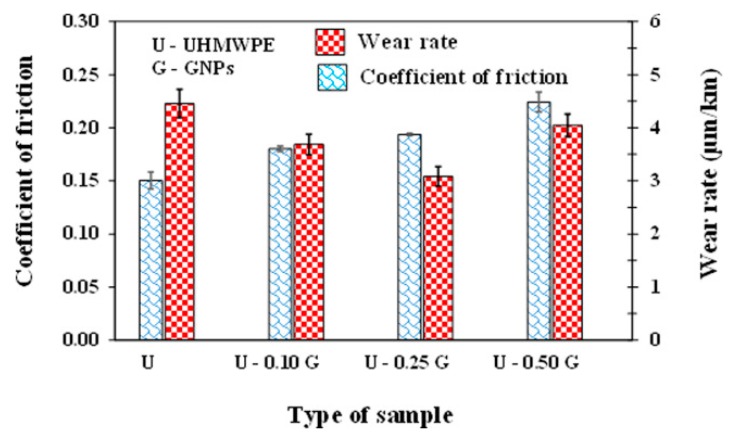
The COF of UHMWPE for different aging times. Reprinted with permission from [134].

Graphene has gained much attention as a filler material in HUMWPE due to its excellent thermal, mechanical and electrical properties [133]. Puertolas [35] concluded that the use of graphene as a filler in UHMWPE should lead to an increase in mechanical properties. The filler content of graphene was found to be very critical for the strengthening of UHMWPE, since fillers enhance mechanical performance at the lowest concentrations; but after this preliminary stage, the material showed a decrease in performance with increasing filler content. Regardless of the graphene product used in nanocomposites, mechanical properties reach the best values at an optimal filler concentration, which is not always the same for the different mechanical parameters. Aliu [134] developed graphene nanoplatelets (GNPs)/UHMWPE composites for improving tribological properties. The 31% reduction in wear rate was observed as compared to pure UHMWPE with addition of 0.25 wt.% GNPs particles in UHMWPE. The friction response of GNPs/UHMWPE composites tested at 0.1 m/s velocity and 8 MPa Pressure is shown in Figure 8. The COF increased with the addition of GNPs up to 0.24. This can be attributed to the anchoring of the UHMWPE chains by GNPs preventing them from sliding over each other. Alam [135] studied the influence of GNPs in UHMWPE. With increase in crystallinity and increase in elastic properties, a significant increase in electrical properties at 3–10 wt.% GNP concentration of GNP/UHMWPE composite exhibit that such composites are useful for smart biomedical implants. Moreover, the addition of graphene into UHMWPE enhanced the thermal stability as compared to the pure UHMWPE [105].

The concentration, orientation and distribution state of carbon nanoparticles are key aspects in the reinforcement of the UHMWPE [136,137]. The dispersion of carbon nanoparticles inside the UHMWPE is a challenge due to its high melt viscosity. Baena [138] studied the effect of the dispersion state of multi-walled CNTs into the UHMWPE on tribological and mechanical performance. The tribological performance was improved with the variation in the content of multi-walled CNTs, however, this improvement was less obvious due to the defects induced by the multi-walled CNTs’ agglomeration. 

### 3.2. Other Reinforced Particles

The reinforcement with soft particles enhances the viscoelastic behavior of UHMWPE, while the reinforcement with hard particles can effectively enhance the load-carrying ability and improve the wear resistance of UHMWPE. It is very crucial to consider that the filler’s shape, filler’s size, filler’s type, filler–matrix interaction, filler loadings, and filler’s dispersion into UHMWPE and shape are important in determining the wear behavior of UHMWPE composites. The effect of particle reinforcement on crystallinity, tribological and mechanical performance reported in previous studies is presented in Table 3. 

Chang [139] studied the influence of zeolite particles into UHMWPE on tribological and mechanical performance. The elongation at break and tensile strength were reduced, but modulus was increased by adding the different concentrations of zeolite particles. In addition, the COF was also decreased with the incorporation of zeolite. Furthermore, smoother surfaces and shallower grooves were observed with the reinforcement of zeolite. For lubricant film transfer, the counter surface of UHMWPE was rough, partially covered, and discontinuous, while for zeolite/UHMWPE it was smooth, covered and continuous. Overall, the addition of zeolite into UHMWPE showed significant effectiveness for improving tribological performance.

Chen [140] conducted an experimental study on polyimide/UHMWPE composites for developing matched sliding materials. The friction efficiency of the mating pair was decreased with increasing the polyimide concentration, while surface roughness and wear rate. The optimum concentration of polyimide was 50 wt.% in terms of lowest surface roughness and wear rate. The nanoclays are also a very promising filler material due to their good barrier and mechanical properties, particle size, morphology, abundance and low cost. The 1.5 wt.% addition of nanoclay significantly increased wear life of and mechanical properties of UHMWPE [141]. Gurgen [143] found that UHMWPE exhibit good interaction with aramid additive and thus, the reduced wear rates were observed for the aramid/UHMWPE composites. At the manufacturing stage, the microstructural consolidation is increased due to the increased molding pressure and thus provide a wear resistant PTFE/UHMWPE composite. Due to the lower COF of PTFE, the material exhibits lower frictional interaction in the sliding conditions. Shi [144] found that the SiO_2_ nanospheres (SNS)/UHMWPE composites exhibit significant improved tribological properties as compared to pure UHMWPE. Huang [146] reported the results for Alendronate sodium (ALN)/UHMWPE composites. The low COF’s were observed at lower loads, while at higher loads the COF were higher than those of UHMWPE under deionized water and saline.

Chang [147] reported that the incorporation of micro and Nano-ZnO with different filler loadings in UHMWPE matrix can improve the wear behavior of the composites. The UHMWPE composites with 5–10 wt.% of micro-ZnO fillers exhibit the lower weight loss while for Nano-ZnO/UHMWPE composites, 10 wt.% exhibits the lowest weight loss under the sliding speed of 0.033 and 0.368 m/s. The weight loss increases with an increase in applied loads and sliding speeds for bothmicro and Nano-ZnO/ UHMWPE. Prasad [152] compared the effect of content of micro and nano ZnO in UHMWPE and found that UHMWPE with 5% nano ZnO showed lowest wear rate. The smooth worn-out surfaces were observed nano-ZnO/UHMWPE composites as compared to micro-ZnO/UHMWPE composites. 

UHMWPE possess poor processability due to high melt viscosity which is the result of high inter-chain entanglement density [142]. Blending UHMWPE with viscosity polymer is the most common method for improving processability. Many researchers blended UHMWPE with polyethylene, polypropylene [153,154], adding polysilane and paraffin oil [155,156], or adding organic clay, kaolin, organic montmorillonite, etc. [157,158,159]. Among these, polyethylene is suitable due to the better miscibility and structural similarity. Khashoggi blended UHMWPE with HDPE and found that the viscosity, storage modulus, and loss modulus were decreased by increasing HDPE content. The mechanical strength was also decreased by increasing HDPE content. Li [142] blended UHMWPE with PEG and found less chain entanglement level of blended UHMWPE. The incorporation of HDPE improved the processability and mechanical properties of the UHMWPE/PEG composites. The flexural modulus, flexural strength and tensile strength of UHMWPE/HDPE/PEG (60/40/4) were increased by 32.5%, 25.7% and 13.8% respectively compared with UHMWPE/PEG (100/4).

## 4. Surface Modifications

### 4.1. Coating

**Figure 9:**
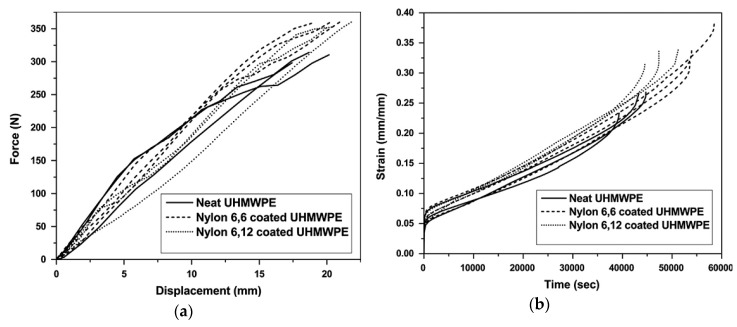
(**a**) Tensile test results of pure and nylon coated UHMWPE fibers at 25 °C (**b**) Creep test results of pure and nylon coated UHMWPE fibers at 25 °C. Reprinted with permission from [160].

The coating of wear-resistant material can enhance the durability of artificial joints. The relation of a surface to coating is important in determining the success of an orthopedic implant. Firouzi [160] performed mechanical tests on nylon coated UHMWPE to investigate the mechanical properties at different temperatures. The results showed the improvement in toughness, braking force, creep time, the maximum braking force for Nylon coated UHMWPE as compared to pure UHMWPE. The results are presented in Figure 9. This research suggests that nylon coated UHMWPE can be employed in biomedical applications due to good mechanical properties, less wear debris and lower cytotoxicity as compared to pure UHMWPE.

Berumen [161], coated the semi-trapezoidal surfaces of UHMWPE with TiAlV film to improve its biocompatibility and mechanical properties. The viscoelastic behavior was decreased, but the load-carrying capability was increased due to the metallic film. The coating detachment and fractures of the metallic film were examined in all scratch tests.

Azam [141] developed the nanoclay reinforced UHMWPE composite coating to improve the tribological properties. A novel electrostatic powder spraying technique was used to deposit the coatings on an aluminum substrate. The results showed that 1.5 wt.% nanoclay/UHMWPE coating was failed at higher cycles as compared to the pristine UHMWPE coating which failed at lower cycles under the same conditions. The enhancement in the performance of 1.5 wt.% nanoclay/UHMWPE coating is attributed to the resulting exfoliated topography of the nanoclay platelets into the UHMWPE matrix due to its homogeneous dispersion that provides an efficient bridging effect, holding the polymer chains together and resisting their easy pull-out. 

The results reported in the literature on the influence of coating for improving tribological and mechanical performance are given in Table 4. The results showed that coating of significantly improved the mechanical properties.

### 4.2. Surface Texturing 

The contact region of the mating pair can be significantly reduced by fabricating proper and well-defined texturing. The lubrication state is shifted from boundary lubrication to full lubrication [166]. The amount of trapped lubricants within the grooves increases and friction reduces due to the load-carrying capability of lubricant. Several texturing methods such as chemical etching [167], pellet-pressing [168], electrochemical micromachining [169], laser surface [170], diamond embossing [171], and electric discharge [172,173], etc., have been employed for texturing on mechanical components.

Ippolito established a procedure to texture UHMWPE components using a novel die in a hot embosser. Four processing parameters chamber temperature, hold duration, hold pressure and cooling were varied to produce textures in specified dimensions. The 96% of the desired texture diameter was achieved on 1800 N hold pressure, 100 °C hold temperature, 20 °C cooling at 1 °C/min rate and 60 minutes hold duration.

Wang et al. [174] examined the load-carrying capacity for textured SiC thrust bearing sliding in water. Texturing in the shape of micro-pits, uniformly distributed in square arrays was done by reactive ion etching on one of the contact surfaces. The existence of optimum micro-pits texture distribution was found, where we can increase load carrying capacity twice as compared to the un-textured surface. Zhou et al. [175] investigated the influence of geometric shape and orientation of dimples and significant effect of such parameters on load-carrying capacity was observed. Rapoport [176] produced the micro-textures by laser texturing to study the influence of texturing on tribological performance under of surfaces under solid lubricating conditions. The influence of bulges height and dimples density were studied. 40–50% dimples density is revealed as optimum density. Surfaces lapped to half of the height of bulges showed the best result. Amanov et al. [177] compared the dimpled rim specimen with bulges with the polished specimen and concluded that the friction and wear rate of dimpled surfaces with bulges was lowest as compared to the polished surfaces probably due to the reducing the contact area. 

**Figure 10:**
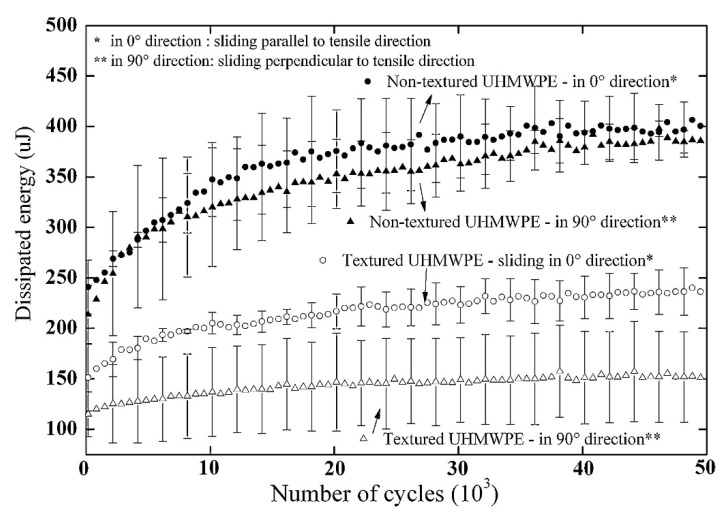
Plots of dissipated energy versus sliding cycles for textured and un-textured UHMWPE. Reprinted with permission from [17].

Zhang [178] studied the influence of textures on UHMWPE and compared them with steel surfaces. The UHMWPE textured surfaces were more effective in terms of lowered frictions as compared to steel surfaces at high loads. The optimum texture density for UHMWPE was in the range of 16% to 30%. Taylor [179] studied the influence of the lubrication regime under varying load and speed conditions to reduce the friction of UHMWPE. The results showed that 50% of COF reduced for textured surfaces as compared to un-textured UHMWPE. Eddoumy [17] examined the effect of the textures on the tribological performance of UHMWPE under reciprocating sliding tests. The two times increase in roughness was confirmed for textured specimens. However, a decrease in dissipated energy was observed for textured UHMWPE as compared to un-textured UHMWPE. Plots of dissipated energy versus number of sliding cycles are shown in Figure 10. Considering that wear resistance increases with decreasing dissipated energy, textured UHMWPE may have anti-wear properties.

Riveiro [180] studied the influence of several laser texturing conditions such as scanning speed, pulse frequency, spot overlapping, and irradiance on texturing. The effect of these processing conditions has been determined to control the melt viscosity altering the cell-material interaction.

Zhang [181] performed a numerical study to compare the different textured geometries including triangle, circle, rectangular and square. The influence of area density, depth and dimple radius on frictions were investigated and optimum parameters were determined. The results showed that the square textures exhibit the lowest frictions due to the highest area density. The results for the effect of texturing on UHMWPE reported in previous studies are given in Table 5. 

## 5. Conclusions

Irradiation is the most common method for sterilizing and/or crosslinking UHMWPE. The free radicals produced by these methods create the inter-chain covalent bonds, leading to the formation of crosslinking. The diffusion of free radicals out to polymer matrix or diffusion of oxygen into the polymer as a result of irradiation can lead to the development of a subsurface oxidized band. This subsurface oxidation region can lead to delamination and in many cases, failure occurs from subsurface crack initiation and propagation. 

The results showed that all radiation-induced cross-linked UHMWPE exhibits high wear performance, but the oxidation reduces the mechanical performance of UHMWPE. The dose and dose rate of irradiation strongly influence the crystallinity and oxidation of UHMWPE. The transvinylene content and crosslink density increased at a higher radiation dose. The variations in the level of crosslinking, crystallinity and oxidation change the wear and mechanical performance. The other parameters, such as temperature, packaging atmosphere and packaging, processing conditions, also influence the distribution and the amount of the oxidation products. With aging, oxygen diffuses deep into the UHMWPE components, reacts with trapped free radicals, and eventually causes more oxidation. 

Subjecting the radiated polymer to a suitable post-irradiation-induced crosslinking process eliminates these radicals. A subsequent remelting step eliminates free radicals to promote oxidative stability and to prevent UHMWPE degradation over the long term. Sterilizing UHMWPE in oxygen-depleted atmospheres, like vacuum packaging or inert gas, can reduce the degree of oxidative degradation. Vitamin E has been considered an important antioxidant to reduce the oxidation and wear degradation of the UHMWPE component. Vitamin E reacts with trapped free radicals into the UHMWPE, impending them to react with oxygen. Thus, it prevents oxidative degradation of UHMWPE and increases its resistance to wear and fatigue.

Another important method to enhance the properties of UHMWPE is the reinforcement with other particles. Carbon nanoparticles are a promising additive for enhancing wear resistance and mechanical properties of UHMWPE. The distribution state and concentration of carbon nanoparticles are predominant factors in the reinforcement of the ultra-high molecular weight polyethylene (UHMWPE). Due to the high melt viscosity of UHMWPE material, the incorporation of carbon nanoparticles into UHMWPE is greatly complicated and homogenous dispersion is a challenge. Many other particles such as Nano ZnO, Fe-Al_2_O_3_/EVA, Alendronate sodium (ALN), SiO_2_ nanospheres, Talc, Zeolite, Nanoclay, aramid, Hydroxyapatite (HA), High-density polyethylene (HDPE), Polyimide, Polytetrafluoroethylene, Polyethylene glycol (PEG) have been reported in the literature for enhancing properties of UHMWPE. The fraction, size, dispersion method, etc., strongly influence the wear and mechanical properties of UHMWPE. 

The durability of artificial joints can be enhanced with a highly wear-resistant coating. The relation of a surface to coating is important in determining the success of an orthopedic implant. The literature showed that coating the surface of UHMWPE enhanced the mechanical properties but can reduce the wear-resistance. The selection of coating material is an important consideration for this purpose. The results reported in the literature related to the texturing of UHMWPE showed that texturing is an important method to enhance the friction and wear resistance of mating surfaces. However, there is a need to study the influence of texturing on mechanical properties. 

## Figures and Tables

**Table 1:** Average properties of Ultra-High Molecular Weight Polyethylene (UHMWPE) and high-density polyethylene (HDPE). Reprinted with permission from [7].

Property	UHMWPE	HDPE
Melting temperature (°C)	132–138	130–137
Molecular weight (10^6^ g/mol)	3.5–7.5	0.05–0.25
Specific gravity	0.925–0.945	0.952–0.965
Poisson’s ratio	0.46	0.40
Modulus of elasticity (GPa)	0.5–0.8	0.4–4.0
Tensile ultimate strength (MPa)	39–48	22–31
Tensile yield strength (MPa)	21–28	26–33
Tensile ultimate elongation (%)	350–525	10–1200
Degree of crystallinity (%)	39–75	60–80
Impact strength (J/m of notch)	1070	21–214
Wear Rate (mm^3^/10^6^ cycles)	80–100	380–400

**Table 2:** Influence of irradiation on crosslinking, tribological and mechanical performance for UHMWPE.

Ref.	Radiation Source	Radiation Dose/Optimum Value	Crystallinity/Crosslinking	Tribological Results	Mechanical Results
[96]	Gamma	50–255 kGy/50 kGy			Impact Toughness-67% Tensile Toughness-64% Elongation-74%
[91]	Gamma		Gel content->650%	Wear rate-35%	
[60]	Electron	25–200 kGy/50 kGy	Crystallinity-110%		Oxidation Index-110%
[45]	Electron	25–100 kGy/25 kGy	branching in 1,7-octadiene-570%		Ultimate Tensile Stress-111% Elongation at break-89.25%
[85]	Gamma	25 kGy	Cross-linking (%)-228% crystallinity-105%	Wear loss-150%	Oxidation index-225%
[87]	Gamma Gas plasma	25 kGy			Tension fatigue-Crack inception Gamma air-88% Gamm inert-86% Gas Plasma-99%
[5]	^60^Co	35 kGy	Crystallinity-119%		
[95]	Gamma	25–40 kGy	Crystallinity (%)->116%		Elastic modulus-273% Peak Load-90% Ultimate load-41%
[55]	Gamma irradiated in N_2_ and air	25 kGy, 50 kGy, 100 kGy/100 kGy at 2.5 k Gy/h dose rate	Gel content (%)-164% Extract fraction (%)-27% Swell ratio-24%	Relative wear rate-140% at 50 kGy	Oxidation index-200% Trans-vinylene index-112% At 25 k Gy/h the values are lower
[59]	Electron-beam	50, 75 &100 kGy/50 kGy	Crosslink density (dm^3^/mole)-116%		Tensile strength (MPa)-103% Toughness-82% Elongation-83% Transvinylene index-102%
[97]	Gamma	35 & 70 kGy/70 kGy			Tensile modulus-86.6% Tensile strength-95.4% Hardness-103.6% Elongation at break-58.1%
[39]	gamma	33-500 kGy/14.5 Mrad	Crystallinity %-126.5% Crosslink density-<747%	Wear rate->6%	Impact of strength-50% Hardness-100% Tensile strength-87% Elongation at break (%)-61%

**Table 3:** Influence of particle or fibers reinforcement on crystallinity, tribological and mechanical results as compared to UHMWPE.

Ref.	Reinforced Particle	Concentration/Size	Crystallinity	Tribological Results	Mechanical Results
[140]	Polyimide	10–90 wt.% Optimum-50% wt.%	Increase in crystallinity and stability	COF-65–75% Wear rate-15%	-----
[127]	Nano-diamond	0.5, 1 & 2 wt.% 30–40 nm Optimum-1 wt.%	97.8%	COF-76% Wear rate-28%	Yield Stress-No change Micro Hardness-97.6%
[126]	Carbon Nanotubes	0.1, 0.45 & 0.5 wt.%, Optimum-0.1 wt.%	3% decrease in melting peak.	Wear rate-118%	Micro Hardness-100.2%
[139]	Zeolite	10 wt.% 20 wt.% Optimum-10 wt.%	-----	COF-approx. 80–90% Volume loss-approx. 80–85%	Tensile Strength-89% Impact Strength-125% Modulus-131% Elongation-89.2%
[126]	Nacre coated with PFPE	12 wt.%	12% reduction in melting peak	Wear rate-251%	Micro Hardness-114%
[132]	Carbon Fibers	Variations in no. of layers Optimum-CF/UF/CF-2/12/2			Flexural Strength-509% Flexural Modulus-284% Ballistic Limit-91%
[141]	Nanoclay	0.5, 1.5 & 3 wt.% Optimum-1.5 wt.%		Wear Life- greater than 10,000 cycles	Hardness-134%
[138]	Multi-walled carbon nanotubes	0.1, 0.5 & 1 wt.% Optimum-1 wt.%		COF-approx. same Wear Rate-74%	Hardness-105%
[142]	Polyethylene glycol (PEG)	Best UHMWPE/PEG ratio 60/4		Shear viscosity-33.3% Storage Modulus-25.5% Loss of modulus-68%	Flexural strength-79.8% Flexural Modulus-77.5%
[135]	Graphene nanoplatelets (GNP)	0.1 wt.% to 10 wt.% Optimum-0.5 wt.%	Crystallinity %-103%		Elastic modulus-130% Yield strength-113% Tensile strength-75% Toughness-76%
[143]	Aramid	2, 3 & 5 wt.%		Roughness-172% Specific wear-60% COF-107%	Hardness-700%
[143]	Poly-tetra-fluoro-ethylene	2, 3 & 5 wt.% Average		Roughness-159% Specific wear-83% COF-91%	Hardness-500%
[124]	High density polyethylene (HDPE)	20, 40, 50, 60, 80 wt.% Optimum- 50 wt.%			Tensile yield stress. 86.3% Tensile strength-69.5% Strain at the break. 380%
[144]	SiO_2_ nano-spheres	0.5, 1, 2, 4 wt.% Optimum-1 wt.%	Degree of crystallization %-96%	COF-50% Volume wear rate-29.4% Mass wear rate-90%	
[145]	Fe-Al_2_O_3_/vinyl acetate (EVA)	18 wt.% of EVA, <50 nm size of Al_2_O_3_ 1, 3 & 5 wt.% of Fe-Al_2_O_3_ Optimum-1 wt.% of Fe-Al_2_O_3_			Tensile Strength-200.7% Modulus of Elasticity-139.3%
[146]	Alendronate sodium (ALN)	1.0 wt.%		COF-approx. 90% Specific wear rate- approx. 110%	Young’s Modulus-97.5% Micro-hardness-96.8% Tensile strength-84.4%
[147]	Nano ZnO	5–20 wt.% Optimum-10 wt.% Size-<100 nm		Wight loss (mg)-58.5% COF-100%	
[148]	Carbon Fibers (CF)	5–30 wt.% Optimum-20 wt.%		COF-139% & 220% Wear Volume-20% & 35%	Hardness-140%
[149]	Hydroxyapatite (HA)	4.7–22. wt.% Optimum-22.8 wt.%			Modulus-888% Yield strength-104% Elongation at break-74%
[150]	kaolin	Size-10 µm 11–26.5 wt.% Optimu-20 wt.%		COF-87% Wear rate-56%	
[105]	Graphene	0.5–3 wt.% Optimum-0.7 wt.%	Degree of crystallization (%)-101%		Linear weight loss temperature-102 % Micro-hardness-110% Toughness-55%
[151]	Talc	10 & 20 wt.% Optimum-20 wt.%	Degree of crystallization (%)-108%		COF-55% Wear rate-50%

**Table 4:** Effect of coating on tribological and mechanical performance.

Ref.	Coating materials	Thickness	Tribological Results	Mechanical Results
[162]	Polypyrrole/Carbon nanotubes			Nominal compressive transverse modulus-500% Bending Modulus-147% Bending rigidity-515%
[163]	Hydrogenated diamond-like carbon (HDLC)	250 nm and 700 nm	COF-200% Wear rate-85%	Nano-hardness-200%
[161]	TiAlV	4.59 µm	Wear rate-118%	Surface hardness-35% at lower load while 200% at higher load
[164]	Nylon 6, 12	0.53 mm		Static load resistance-186% Energy absorption-145 to 316%
[165]	Poly(methyl methacrylate)—hydroxyapatite (PMMA/HA)	32.61–34.01 µm	COF-75% Wear rate-65%	

**Table 5:** Effect of texturing on tribological performance.

Ref.	Texturing Method	Shape	Area Density	Tribological Results
[178]	Photolithography and electrolytic etching	Round dimple	5–40% Optimum-30%	COF-76% Wear depth-64%
[182]	Nanoimprint lithography (NIL)	Rectangular grading array	Area density-50%	Static friction-43–55% COF-60–90%
[181]	Numerical	Circular, Rectangular, squared &Triangular	Circular-26%, Rectangular-17% squared-20%, Trianglular-21%	COF for Circular-89.1%, Rectangular-71.9% squared-71.4%, Trianglular-74.6%
[183]	Laser surface	Squared Rectangular	Squared-51% Rectangular-43.4%	COF-45% Wear track depth-71%
[179]	Micromachining	Dimple	3.1, 12.6, 50.2% Optimum-12.6%	COF-50%
